# Anodal transcranial direct current stimulation prevents methyl-4-phenyl-1,2,3,6-tetrahydropyridine (MPTP)-induced neurotoxicity by modulating autophagy in an *in vivo* mouse model of Parkinson’s disease

**DOI:** 10.1038/s41598-018-33515-7

**Published:** 2018-10-11

**Authors:** Sang-Bin Lee, Hee-Tae Kim, Hyun Ok Yang, Wooyoung Jang

**Affiliations:** 1 Natural Medicine Center, Korea Institute of Science and Technology, Gangneung, 25451 Republic of Korea; 2 School of Pharmacy, Sungkyunkwan University, Suwon, 16419 Republic of Korea; 3 Department of Neurology, Hanyang University College of Medicine, Seoul, Republic of Korea; 4 Division of Bio-Medical Science &Technology, KIST School, Korea University of Science and Technology, Seoul, 02792 Republic of Korea; 5 Department of Neurology, Gangneung Asan Hospital, University of Ulsan College of Medicine, Gangneung, Republic of Korea

## Abstract

Parkinson’s disease (PD) is a neurodegenerative disorder characterized by the accumulation of protein inclusions and the loss of dopaminergic neurons. Transcranial direct current stimulation (tDCS) is a non-invasive brain-stimulating technique that has demonstrated promising results in clinical studies of PD. Despite accumulating evidence indicating that tDCS exerts a protective effect, the mechanism underlying its activity remains unknown. In the present study, we first investigated the neuroprotective effect of tDCS in a 1-methyl-4-phenyl-1,2,3,6-tetrahydropyridine (MPTP)-induced PD mouse model and then evaluated the effect of tDCS on the autophagy pathway. tDCS improved behavioral alterations, increased tyrosine hydroxylase protein levels and suppressed α-synuclein protein levels in MPTP-treated mice. MPTP-treated mice subjected to tDCS also had lower levels of autophagy-related proteins, such as microtubule-associated protein 1 light chain 3 and AMP-activated protein kinase, and higher levels of mechanistic target of rapamycin and p62. In addition, the protein levels of phosphoinositide 3-kinase and brain-derived neurotrophic factor were higher, and the levels of unc-51-like kinase 1 were lower in MPTP-treated mice subjected to tDCS. Our findings suggest that tDCS protected against MPTP-induced PD in a mouse model by modulating autophagy.

## Introduction

Parkinson’s disease (PD) is currently defined as a neurodegenerative disorder characterized by the progressive loss of dopaminergic (DA) neurons in the substantia nigra pars compacta (SNpc). Its pathologic hallmark is Lewy bodies. Currently, the management of PD is mainly focused on pharmacologic treatments, such as L-3,4-dihydroxyphenylalanine (L-DOPA) or dopamine agonists, which improve the clinical symptoms of PD^1^. Although symptomatic treatment has been shown to increase quality of life in PD patients, no disease-modifying treatment that eventually reverses or stops the disease’s progression is currently available, and the development of a neuroprotective therapy is one of the greatest unmet goals in PD. Furthermore, longstanding pharmacological treatments can result in undesirable adverse effects, such as motor fluctuations and L-DOPA-induced dyskinesia, as the disease progresses^2^. Therefore, it is essential that an alternative treatment modality that is safe and effective is developed to manage PD patients.

Transcranial direct current stimulation (tDCS) is a non-invasive neuromodulatory technique that acts by stimulating cortical and subcortical structures in the brain and modulating cortical excitability by inducing a constant but weak current between two electrodes^3^. While many recent studies have shown that tDCS potentially improves and enhances cognitive and behavioral functions in addition to motor functions in PD patients, the results of tDCS, when applied in PD, have varied^4–7^. Furthermore, Lu *et al*. recently reported that tDCS exerted a neuroprotective effect in a 1-Methyl-4-Phenyl-1,2,3,4, Tetrahydropyridine (MPTP)-induced parkinsonian mouse model by reducing oxidative stress markers^8^. Although the specific mechanism underlying this process remains unclear, the cellular and molecular effects of tDCS also include anti-apoptotic and anti-inflammatory effects and the capacity to alter neurotransmitters^9,10^.

Autophagy is a lysosome-mediated catabolic pathway that is responsible for the bulk and non-specific degradation of cytosolic proteins and organelles, which contributes to the maintenance of cellular homeostasis^11^. Many lines of evidence have indicated that autophagy plays an important role in the pathogenesis of PD. Furthermore, the pathogenesis of PD encompasses a combination of several biochemical factors, including oxidative stress, mitochondrial dysfunction, excitotoxicity, and inflammatory oxidative stress, all of which are tightly linked to autophagy pathways and several studies showed that tDCS could affect reactive oxygen species, apoptic pathway and neuroinflamation^8,9,12^. Therefore, if tDCS exhibits a neuroprotective ability against neurodegenerative processes in a PD model, the mechanism responsible for its activity may involve the modulation of autophagy, which could be potential target for disease modifying treatment in PD. In addition, it suggests it is reasonable to investigate whether tDCS could affect the autophagy-lysosomal pathway. However, no previous study has investigated the effect of tDCS on autophagy pathways.

MPTP enters astrocytes and is converted into the active metabolite MPP^+^ by monoamine oxidase (MAO-B). M MPP^+^ subsequently accumulates in dopaminergic neurons, in which it has been shown to inhibit complex I of the respiratory chain of the inner mitochondrial membrane, resulting in the degeneration of dopaminergic neurons and the production of parkinsonian syndromes that are very similar to those observed in PD patients^13^. In a previous study, we demonstrated that MPTP increased LC3-II and decreased SQSTM1/p62 expression, indicating that MPTP activates the autophagy-lysosomal pathway^14^. Su *et al*. also reported that MPTP enhanced aberrant autophagy and that treatment with melatonin restored the excessive activation of autophagy and attenuated MPTP-induced neurotoxicity^15^. Therefore, we hypothesized that anodal tDCS may decrease MPTP-induced neurotoxicity in dopaminergic neurons in an *in vivo* model of PD and that the mechanism underlying its activity may involve the modulation of activated autophagy signaling pathways. We hypothesized that it might thereby improve the behavioral dysfunctions observed in an MPTP-induced mouse model of PD.

In the current study, as the intervention technique, we applied anodal tDCS to the M1 area at an intensity of 0.1 mA. We evaluated molecular markers of neurodegeneration, such as tyrosine hydroxylase and α-synuclein, in addition to behavioral outcomes after we applied anodal tDCS to untreated and MPTP-induced mouse models. Furthermore, we also evaluated the levels of the autophagy-related protein to determine the effect of anodal tDCS on autophagy.

## Results

### tDCS ameliorates motor dysfunction in a mouse model of MPTP-induced toxicity

**Figure 1:**
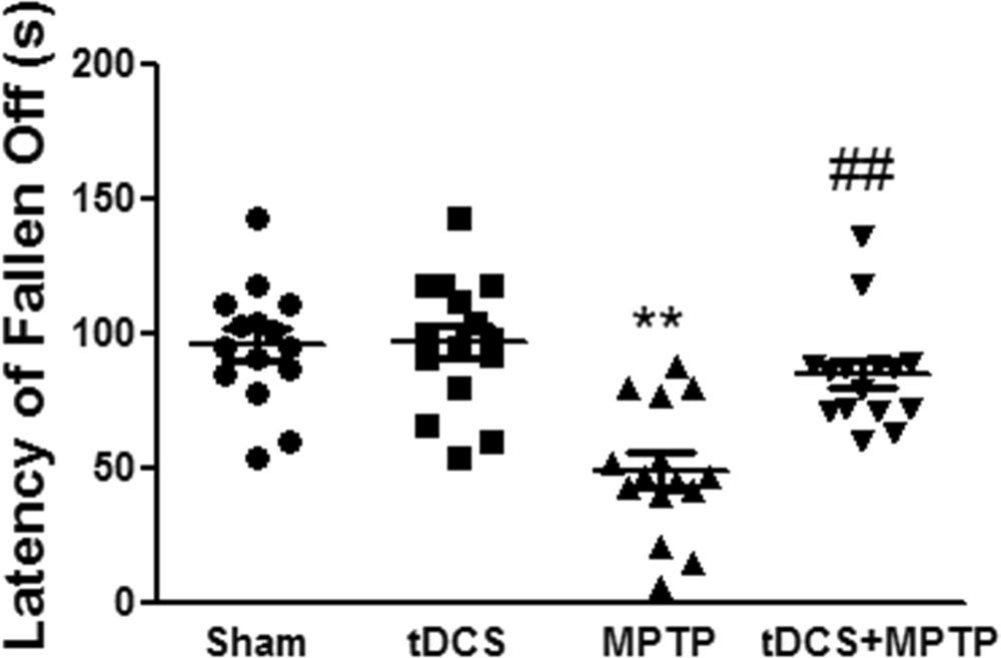
Effect of tDCS on motor dysfunction in MPTP-treated mice. The length of time on the rotarod was recorded (n = 15). All values are shown as the mean ± S.E.M. ^**^p < 0.01 compared to the sham group, ^##^*p* < 0.01 compared to the MPTP group.

To investigate the effect of tDCS on MPTP-induced motor dysfunction, we used the rotarod test. The retention time was 95.73 ± 5.73 seconds in the sham group. After treatment with MPTP, retention times were significantly shorter (approximately 49% of the level observed in the sham group). However, retention times were longer in the MPTP + tDCS group (1.7-fold the times observed in the MPTP-only group, 2-way ANOVA, tDCS: F = 9.531, *P* = 0.0031; MPTP: F = 25.05, *P* < 0.001; interaction: F = 8.641, *P* = 0.0048) (Fig. 1).

### tDCS improves neuroprotection in a mouse model of MPTP-induced toxicity

**Figure 2:**
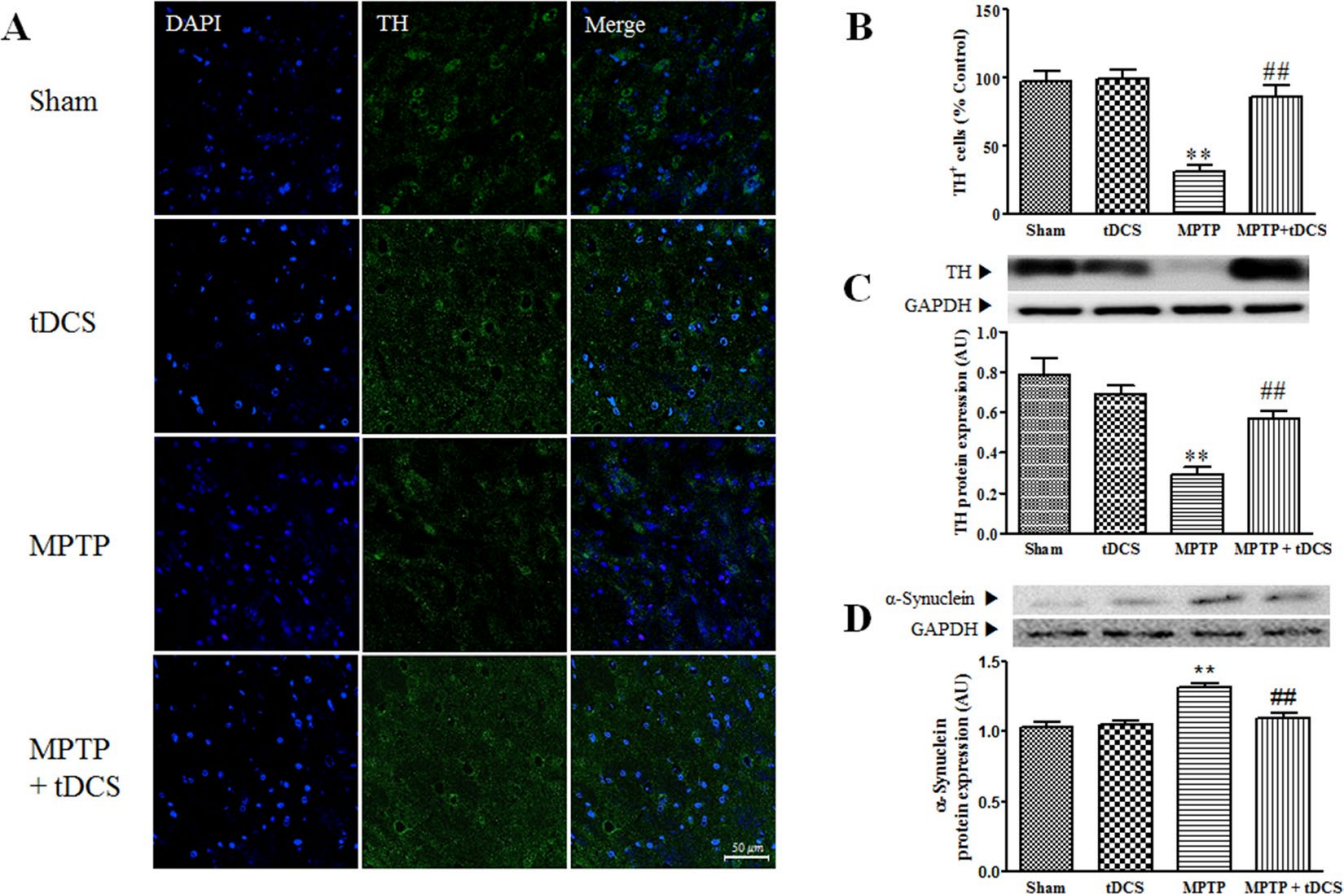
Effect of tDCS on dopaminergic neuronal loss in MPTP-treated mice. After behavioral impairment, dopaminergic neurons were identified using TH-immunocytochemistry. Representative images show the SNc in each group (**A**). TH-positive cells were counted (**B**) in the SNc (n = 6). The protein level of TH in the SNpc was detected using western blotting (**C**) (n = 8 to 9). The level of the α-synuclein protein in the SNpc was determined using western blotting (**D**) (n = 8 to 9). All values are shown as the mean ± S.E.M. ^*^p < 0.05 and ^**^p < 0.01 compared to the sham group, ^#^*p* < 0.05 and ^##^*p* < 0.01 compared to the MPTP group.

Consistent with the results of the motor function tests, tDCS protected dopaminergic neurons against MPTP-induced toxicity. As shown in Fig. 2A,B, there were significantly fewer TH-positive cells (dopaminergic neurons) in the MPTP group (68%) those in the sham group (considered 100%, 2-way ANOVA, tDCS: F = 16.52, *P* < 0.001; MPTP: F = 31.46, *P* < 0.001; interaction: F = 14.47, *P* = 0.0011). Furthermore, the expression of the TH protein was significantly lower in the MPTP group (approximately 63%) than in the sham group (considered 100%). These decreases were attenuated by tDCS (2-way ANOVA, tDCS: F = 3.041, *P* = 0.0911; MPTP: F = 36.82, *P* < 0.001; interaction: F = 13.04, *P* = 0.0011) (Fig. 2C). The expression of α-synuclein protein was higher in the MPTP group, and tDCS attenuated the expression of the α-synuclein protein (2-way ANOVA, tDCS: F = 9.001, *P* = 0.0052; MPTP: F = 23.55, *P* = 0.0052; interaction: F = 12.91, *P* = 0.0011) (Fig. 2D). Treatment with tDCS alone did not affect the loss of dopaminergic neuron cells.

### tDCS inhibits autophagy in a mouse model of MPTP-induced toxicity

**Figure 3:**
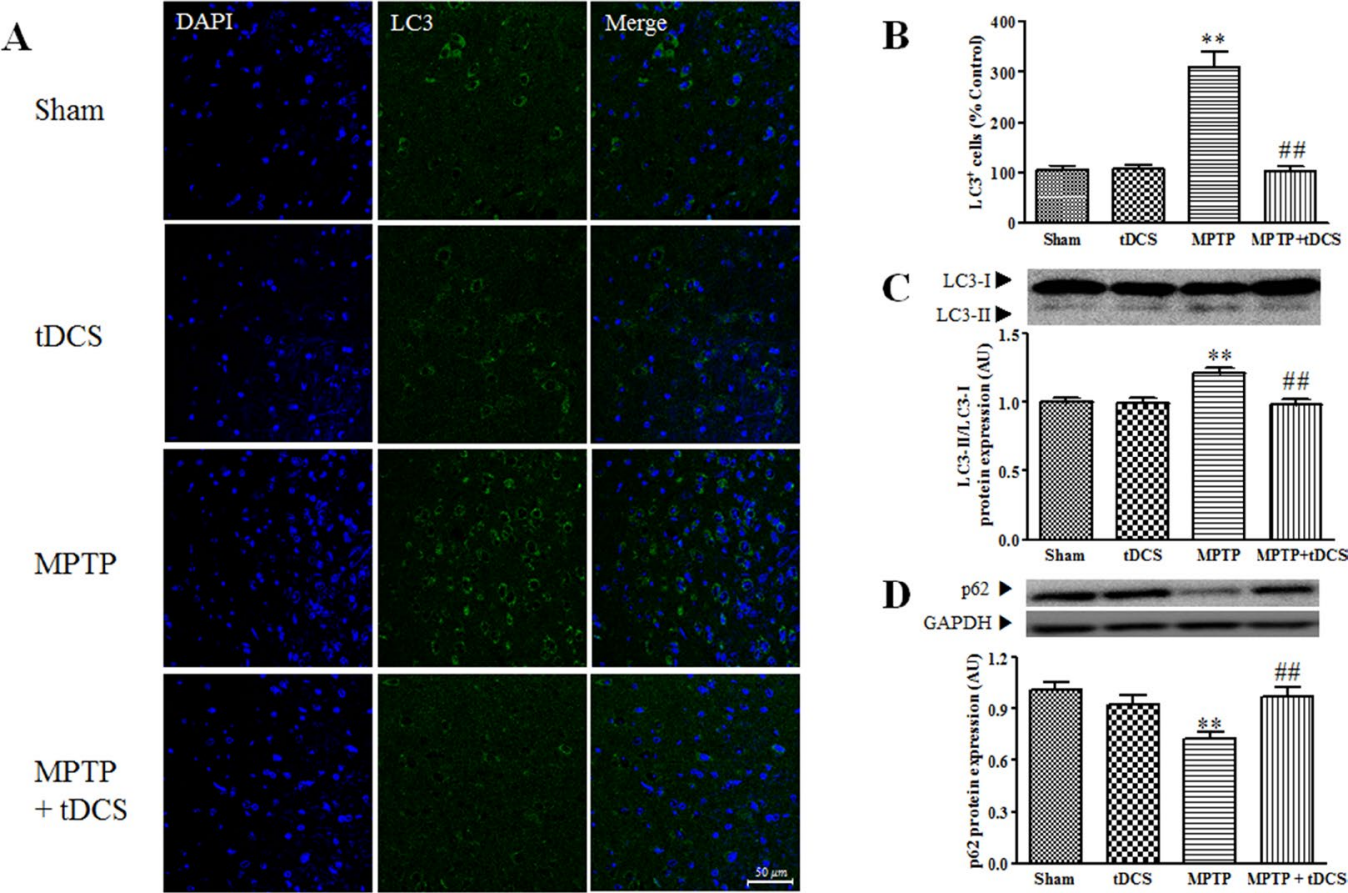
Effect of tDCS on autophagy in MPTP-treated mice. LC3-positive cells were identified using LC3-immunocytochemistry. Representative images show the SNc in each group (**A**) LC3-positive cells were counted (**B**) in the SNc (n = 6). Western blotting was performed to measure the ratio of LC3-II/LC3-I (**C**) and the level of p62 (**D**). The ratio of the LC3-II and LC3-I bands was evaluated by densitometric analysis (n = 9). The expression of p62 protein was adjusted to the level of GAPDH, which was used as the loading control (n = 8 to 9). All values are shown as the mean ± S.E.M. ^*^p < 0.05 and ^**^p < 0.01 compared to the sham group, ^#^*p* < 0.05 and ^##^*p* < 0.01 compared to the MPTP group.

To determine the effects of tDCS on MPTP-induced autophagy, we measured the ratio of LC3-II/LC3-I and the protein expression level of p62 in the mouse SNpc. The ratio of LC3-II/LC3-I was significantly higher in the MPTP group, in which it was 1.2-fold higher than was observed in the sham group (2-way ANOVA, tDCS: F = 10.13, *P* = 0.0032; MPTP: F = 7.068, *P* = 0.0122; interaction: F = 8.978, *P* = 0.0052). In addition, after treatment with MPTP, the expression level of the p62 protein was markedly decreased to approximately 28% of the level observed in the sham group (2-way ANOVA, tDCS: F = 3.225, *P* = 0.082; MPTP: F = 6.507, *P* = 0.0157; interaction: F = 12.10, *P* = 0.0015). TDCS attenuated these changes (Fig. 3C,D). To confirm the results of western blot analysis, we performed LC3-IF in the SNpc. After tDCS, the number of LC3-positive cells was lower than was observed in the MPTP group (2-way ANOVA, tDCS: F = 34.15, *P* < 0.001; MPTP: F = 31.85, *P* < 0.001; interaction: F = 34.93, *P* < 0.001) (Fig. 3A,B).

### tDCS inhibits markers upstream of autophagy in a mouse model of MPTP-induced toxicity

**Figure 4:**
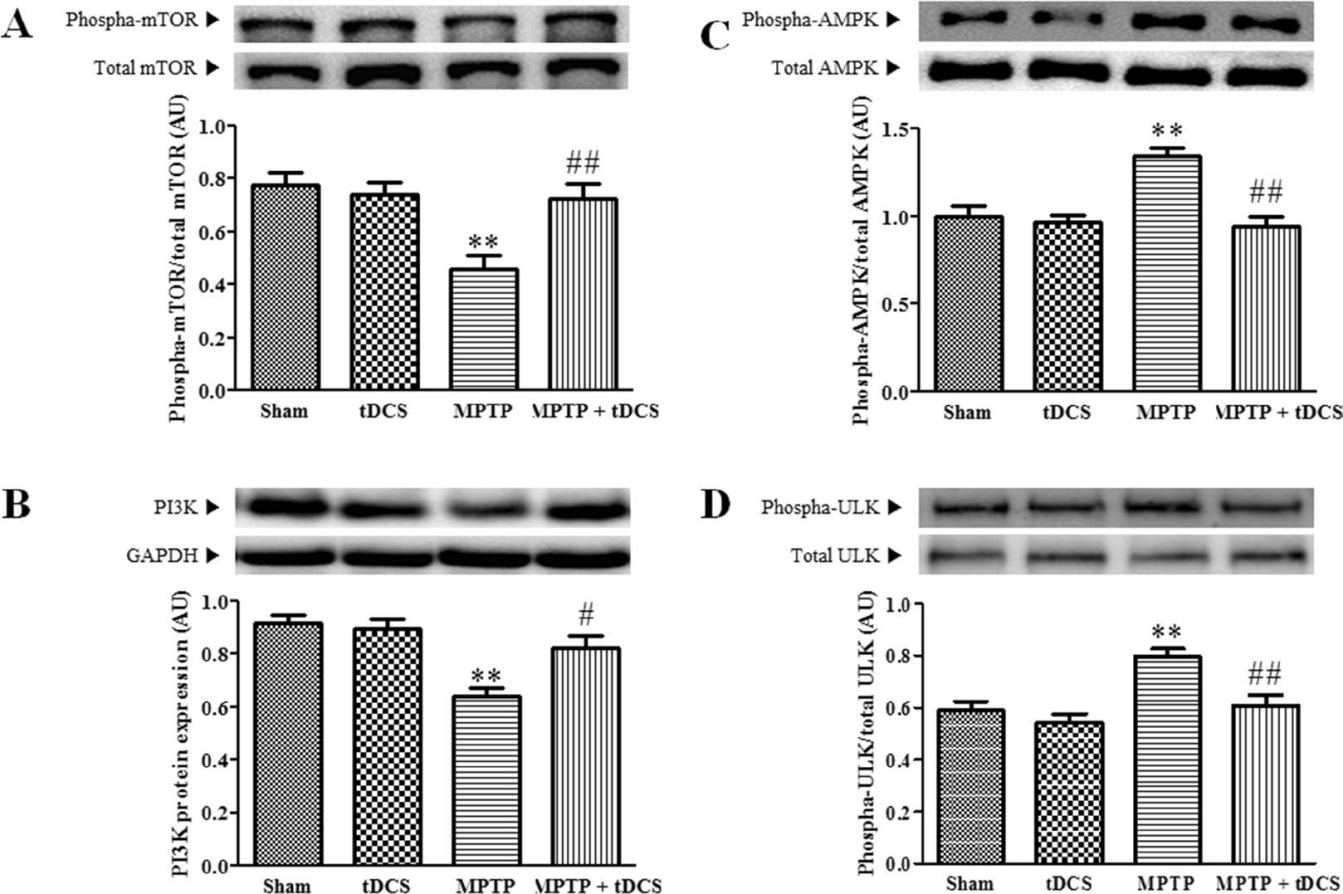
Effect of tDCS on markers upstream of autophagy in MPTP-treated mice. Western blotting was performed to measure the levels of upstream markers of autophagy, including mTOR (**A**) PI3K (**B**) AMPK (**C**) and ULK1 (**D**). The expression of each protein was adjusted to the level of GAPDH, which was used as the loading control (n = 9). All values are shown as the mean ± S.E.M. ^*^p < 0.05 and ^**^p < 0.01 compared to the sham group, ^#^*p* < 0.05 and ^##^*p* < 0.01 compared to the MPTP group.

To examine the pathways upstream of autophagy, we measured the protein expression levels of PI3K, mTOR, AMPK and ULK. After treatment with MPTP, the levels of phosphorylated mTOR (2-way ANOVA, tDCS: F = 5.045, *P* = 0.0317; MPTP: F = 10.53, *P* = 0.0027; interaction: F = 9.146, *P* = 0.0049) and phosphorylated PI3K (2-way ANOVA, tDCS: F = 4.536, *P* = 0.0410; MPTP: F = 20.60, *P* < 0.001; interaction: F = 7.384, *P* = 0.0105) were markedly decreased to 41% and 30%, respectively, of the levels observed in the sham group, and tDCS attenuated both of these decreases (Fig. 4A,B). The protein expression level of phosphorylated AMPK (2-way ANOVA, tDCS: F = 10.01, *P* = 0.0034; MPTP: F = 17.80, *P* < 0.001; interaction: F = 12.68, *P* = 0.0012) and ULK (2-way ANOVA, tDCS: F = 11.25, *P* = 0.0021; MPTP: F = 15.04, *P* < 0.001; interaction: F = 3.9717, *P* = 0.0549) significantly increased to 1.3-fold and 1.4–fold, respectively, of the levels observed in the sham group. These increases were also attenuated by tDCS (Fig. 4C,D).

### tDCS increases the protein level of BDNF in a mouse model of MPTP-induced toxicity

**Figure 5:**
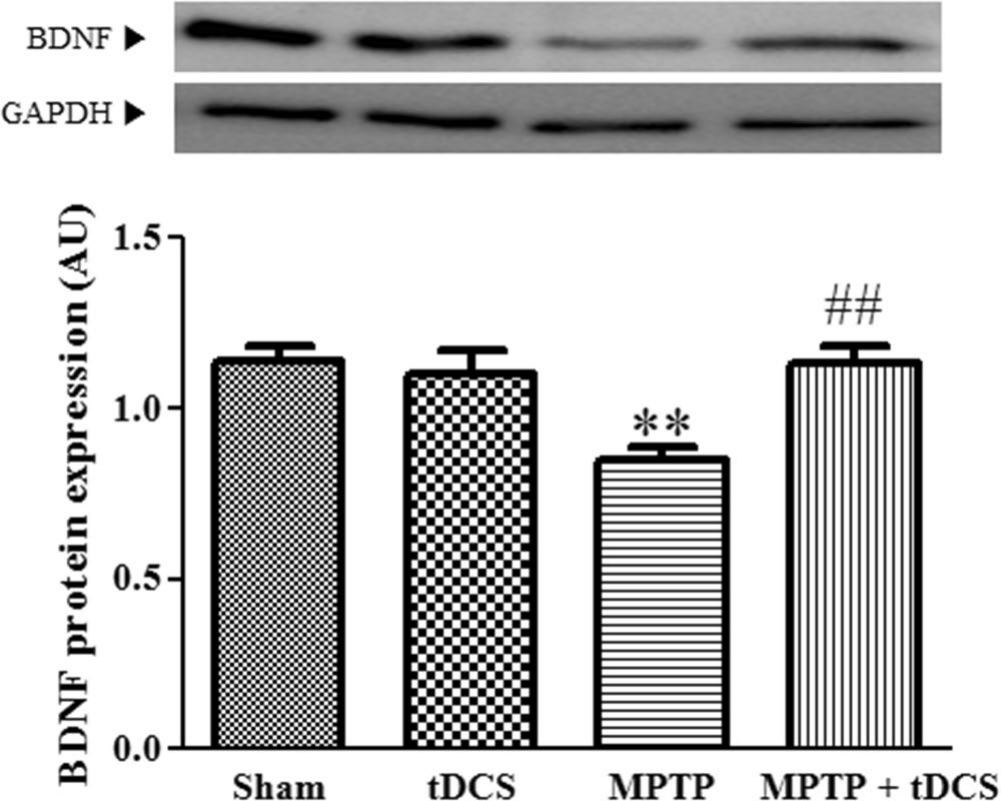
Effect of tDCS on BDNF protein expression in MPTP-treated mice. Western blotting was performed to measure BDNF protein expression levels. The expression of each protein was adjusted to the level of GAPDH, which was used as the loading control (n = 9). All values are shown as the mean ± S.E.M. ^*^p < 0.05 compared to the sham group, ^#^*p* < 0.05 compared to the MPTP group.

To investigate the molecular mechanism by which MPTP and tDCS affect autophagy, we measured BDNF protein expression. We found that the protein expression level of BDNF was decreased to 25% of the level observed in the sham group, and tDCS attenuated this decrease (2-way ANOVA, tDCS: F = 6.059, *P* = 0.0194; MPTP: F = 6.677, *P* = 0.0145; interaction: F = 9.909, *P* = 0.0035) (Fig. 5).

## Discussion

In this study, we demonstrate that anodal tDCS exerts a neuroprotective effect on MPTP-induced dopaminergic neurotoxicity. While applying anodal tDCS in the control group did not significantly change the expression of autophagy-related markers, tDCS downregulated autophagy markers in the MPTP-treated group. Therefore, anodal tDCS might ameliorate the degeneration of dopaminergic neurons in MPTP-induced mice by modulating autophagy. To the best of our knowledge, this is the first study to investigate the effect of tDCS on autophagy-related markers. In our study, LC3II and AMPK were upregulated, while mTOR and p62 were downregulated in MPTP-treated mice, indicating the activation of autophagy-related processes. Anodal tDCS seemed to stabilize the autophagy processes that were activated by MPTP-induced toxicity.

tDCS acts by modifying neuronal excitability. Generally, according to polarity, anodal stimulation enhances cortical excitability, while cathodal stimulation exerts the opposite effect^16^. Fregni *et al*. reported that motor-evoked potentials (MEPs) significantly increased after anodal tDCS was applied in PD patients and that the changes observed in MEPs were correlated with improvements in motor symptoms^17^. However, the cellular and molecular mechanisms by which tDCS exerts its effects in PD patients remain unclear, and few studies have explored its mechanism in animal models of PD. tDCS is known to affect cell migration, orientation, differentiation, and metabolism by shifting intracellular calcium flux, interacting with membrane receptors, including acetylcholine receptors and members of tropomyosin receptor kinase families. N-methyl-D-aspartate receptors (NMDARs) and neurotransmitters, such as gamma-aminobutyric acid (GABA), serotonin, and glutamate, have also been reported to be associated with tDCS-induced long-term potentiation and long-term depression^18^. In particular, BDNF expression was enhanced by anodal tDCS, and this boosted BDNF-dependent synaptic plasticity, which could potentially influence learning and memory^19^. Furthermore, BDNF triggered the expression of PI3K, an enzyme upstream of mTOR that might be responsible for modulating autophagy. Chen *et al*. suggested that BDNF exerted a neuroprotective effect by enhancing autophagy^20^. However, Smith *et al*. suggested that the neuroprotective and autophagy-modulating roles of BDNF could be influenced by mTOR expression^21^. Therefore, the effect of tDCS on autophagy pathways could be dependent on environmental stimulation. In human PD, the efficacy of anodal tDCS has differed between dopaminergic ‘on’ state and ‘off’ state studies. These findings indicate that dopaminergic medications could interact with the mechanism induced by tDCS^22^. In our study, in the group that was not treated with MPTP, the application of tDCS appeared to enhance autophagy but induced no significant changes in autophagy markers. Therefore, tDCS may modulate autophagy in different ways based on the stimulatory environment. Peruzzotti-Jametti *et al*. reported that anodal tDCS enhanced while cathodal stimulation suppressed the expression of the pro-apoptotic marker caspase-3 in a stroke model^23^. The findings that there is a great deal of cross-communication between autophagy and apoptosis pathways and that apoptosis can inhibit autophagy may explain why anodal tDCS modulated autophagy only in the MPTP-treated mice.

In a previous study, Lu *et al*. have reported that anodal tDCS attenuated MPTP-induced toxicity in dopaminergic neurons by reducing oxidative stress, thereby improving behavioral outcomes^8^. Therefore, the anti-inflammatory effect of anodal tDCS could also contribute to the neuroprotective effect observed in our study. Furthermore, autophagy could be initiated by hypoxia or starvation, both of which can affect the expression of autophagy markers^24^. Consequently, the results of our study show that the net effect of anodal tDCS might be to suppress autophagy pathways under oxidative stress conditions, and this effect could ameliorate dopaminergic neurotoxicity. Therefore, the specific mechanism by which tDCS affects autophagy pathways in humang PD subjects remains to be clarified in future studies using sophisticated PD model such as transgenic mouse. Considering that many studies reported autophagy lysosomal pathway was impaired in human PD brains, autophagy pathway modulation could be strong potential mechanism for tDCS efficacy as well as stabilizing oxidative stress^8,12,25^.

MPTP has been shown to upregulate α-synuclein in dopaminergic neurons^15^. In our study, anodal tDCS as also shown to be capable of modulating α-synuclein levels, which are upregulated by MPTP. The protein α-synuclein is a major component of Lewy bodies, which have been identified as a distinctive pathological hallmark of PD. Although the specific functions of α-synuclein remain uncertain, it may be strongly associated with familial and sporadic forms of PD^26,27^. Point mutations in the SNCA gene are responsible for some familial inheritance forms of PD, and polymorphisms in the SNCA gene have also been shown to influence the risk of developing PD. It is possible that genetic and environmental factors induce misfolding in natural α-synuclein and convert it to the toxic oligomeric form that is deposited in Lewy bodies^28^. Therefore, reducing α-synuclein synthesis or increasing its clearance might exert a neuroprotective effect against PD.

In PD, misfolded proteins are degraded via the ubiquitin-proteasome and autophagy-lysosome systems. Many lines of evidence indicate that the autophagy pathway is involved in the pathogenesis of PD and that α-synuclein can be degraded via the macroautophagy pathway^12^. However, in this study, anodal tDCS restored the expression of overactive autophagy-related markers in a PD-like mouse model. Thus, the specific mechanism by which α-synuclein levels are modulated by tDCS remains unclear but might be associated with an alternative pathway. Nonetheless, considering that α-synuclein plays a pivotal role in the pathogenesis of PD, our finding that anodal tDCS can stabilize the MPTP-induced upregulation of α-synuclein in the SNpc indicates that tDCS is a potentially disease-modifying strategy for treating PD.

Several previous studies have shown that tDCS exerts positive effects on behavioral outcomes in animals and humans^3,8,29^. Our result also show that anodal tDCS restores the behavioral deficits induced by MPTP injection. Fregni *et al*. and Coesntino *et al*. showed that treatment with tDCS produced significant improvements in UPDRS-III scores in humans^17,30^. Furthermore, tDCS also had beneficial effects on cognitive function and freezing of gait^31^. Thus, the possibility that tDCS could be used to treat symptomatic patients as well as to provide neuroprotection is very promising. However, till now, there was few clinical trials for proving neuroprotective efficacy of tDCS in human subjects compared to application for candidate of symptomatic treatment and the implementation of this technique awaits further evidence and the development of an ideal protocol to achieve the best results.

There are several limitations to our study. First, we used subacute MPTP model to induce neurotoxicity in our *in vivo* mouse model in mouse of parkinsonism as following previous studies^8,32^. Although this subacute MPTP model is widely recognized and extensively used as an animal model of PD, it does not completely reproduce clinical PD and its progressive nature. Chronic MPTP model which is required repeated MPTP injection over 2 weeks has been introduced and showed more sustained alteration of nigrostriatal cell loss and resemblance of cardinal PD features^33^. Furthermore, various studies have suggested that impaired autophagy pathways, rather than overactive autophagy processes, contribute to the pathogenesis of human PD^12,25^. Therefore, considering that PD is a progressive, chronic degenerative disease, to clarify the role that the modulation of autophagy plays in the neuroprotective effects of tDCS in PD, tDCS should be applied in more elegant PD models, such as chronic MPTP model or transgenic mouse models of PD. Second, the protocol used to perform tDCS is arbitrary. Its polarity, the site of stimulation, and the intensity at which tDCS is applied can greatly affect its effects. For example, in a stroke model, the polarity that was shown to exert a neuroprotective effect as cathodal stimulation^34^. Therefore, cathodal tDCS could be tentative option for neuroprotection in PD model and further investigation using cathodal stimulation is necessary. However, in PD. anodal tDCS seems to be more favorable than cathodal stimulation based on the neurophysiological measurements obtained here and in several previous studies in which tDCS was applied in a PD model or PD patients^8,22^. Furthermore, we initially tested cathodal tDCS stimulation in 3 mice in each group, but found no efficacy on modulating autophagy markers and excluded cathodal tDCS in main experimental process (Supplementary material). With regard for the site of stimulation, application of tDCS at the dorsolateral prefrontal cortex (DLPFC) was also reported to enhance cognitive function and motor performance. Reports have also shown that applying anodal tDCS on M1 may enhance cortical activity via NMDA receptors and prolong the cortical silent period, which reflects dopaminergic function^35^. Furthermore, the M1 area is both directly and indirectly connected with the striatum, and Li *et al*. suggested that the anodal stimulation of M1 also activated the SN^32^. Therefore, there is evidence indicating that M1 is a suitable site for stimulation in PD. However, there are also reports that multitarget tDCS reveal effectively reducing freezing of gait, improving executive function and balance function in PD^36^. Therefore, bi-hemisphetic and multitarget tDCS montage (dorsolateral prefrontal and M1) should be considered for clinical trial in human PD patients andfuture studies will need to develop a protocol that maximizes the neuroprotective efficacy of tDCS. Third, we evaluated only right side of substantia nigra based on previous study. Therefore, effect of anodal tDCS on ipsilateral side remained unclear. Lastly, limited types of autophagy markers were used to evaluate the ability of tDCS to modulate autophagy. We measured the autophagy initiation protein, mTOR, and autophagy-specific cargo, such as LC3II and p62. However, these markers do not precisely reflect autophagy flux. Both enhanced LC3II conversion and reduced autophagosome degradation can cause an increase in LC3II protein levels. Therefore, the precise net effect of anodal tDCS on autophagy flux should be further investigated.

Despite these limitations, our data demonstrate that tDCS exerts a neuroprotective effect against the dopaminergic neurotoxicity observed in an MPTP-induced parkinsonian mouse model and that it modulates α-synuclein expression. The mechanism by which it exerts this neuroprotective effect is likely to involve the modulation of autophagy. These results suggest a theoretical basis for the use of anodal tDCS as a candidate neuroprotective therapy or a therapy to improve symptomatic PD, both of which are of great interest.

## Methods

### Animals

All animal procedures were approved by the Korea Institute of Science and Technology Animal Care and Use Committee (AP-2017009).

Experiments using mice were conducted in accordance with the Korea Institute of Science and Technology Animal Care Committee guidelines and other approved guidelines^37^. Male C57BL/6 mice (7 weeks old) were obtained from Orient Bio Inc. (Seongnam, Korea) and maintained in a temperature- and humidity-controlled room (22 ± 3 °C and 50%, respectively) under a 12-h light/dark cycle. Water and food were provided *ad libitum*.

### Surgery and transcranial brain stimulation

To induce anodal tDCS, we chosen an electrode montage based on earlier studies^8,19,32,38^. Mice were anesthetized with ketamine (55 mg/kg) and xylazine (7 mg/kg), and a small incision was made in the skin of the head. After the skull was dried with cotton swabs, a custom-made plastic tube (American Plastics, CA, USA) was placed on the skull overlying the M1 area of the cortex. The tube was subsequently attached to the skull overlying the frontal cortex by nontoxic dental cement. To avoid debris accumulating in the plastic tube, a custom-made screw cap was placed to seal the tube when not in use. The mice were allowed to recover for five days. The animals (n = 60) were randomly divided into the following four groups: (1) vehicle-treated sham (Sham; n = 15), (2) vehicle-treated anodal tDCS group (tDCS; n = 15), (3) MPTP-treated sham (MPTP; n = 15) and (4) MPTP-treated anodal tDCS group (MPTP + tDCS; n = 15). Mice in the MPTP groups received an intraperitoneal injection of MPTP (30 mg/kg) dissolved in phosphate-buffered saline (PBS) on five consecutive days with or without tDCS. tDCS was daily applied daily for 30 min continuously, using a constant current stimulator (Caputron, NYC, USA) at 0.1 mA by an anodal electrode, 30 min after treatment with MPTP (current density of stimulus electrode: 3.2 mA/cm^2^). The anodal electrode [3.1 mm^2^] was inserted into the tube placed on the left M1 area of the cortex, which was filled with saline, and the cathode [11 cm^2^] was placed between the shoulders. After the last tDCS was applied, all of the mice were submitted to a motor coordination test and then sacrificed. The brain of each mouse was quickly taken and than was fixed in 4% paraformaldehyde for immunocytochemistry or stored at −80 °C for western blotting.

### Motor Coordination Measurements

The rotarod test was performed to assess fine motor coordination and balance after treatment with MPTP in a manner similar to that described in previously published studies^39^. On the three consecutive days before testing, the mice were pre-trained to stay on a rotarod that accelerated to increasing speeds (2–16 rpm) for 3 min 3 times per day. On the test day, we performed three trial with the same procedure. The latency times until the mice fell were automatically recorded, and the resulting data were used to calculate the mean time over the three trials.

### Immunocytochemistry

Midbrain tissues (6 mice per group) were fixed in 0.1 M sodium phosphate buffer containing 2.5% glutaraldehyde and 4% paraformaldehyde (pH 7.2). Serial coronal sections (30 μm thick) were cut through the SNpc on a freezing cryostat (Thermo Fisher, MA, USA). The sections were washed with PBS and placed in 0.5% Triton X-100 in PBS for 30 min to permeabilize the tissues. After the sections were washed with PBS, they were blocked with protein block serum-free buffer for 30 min and then incubated in blocking buffer containing primary antibodies for 2 h at room temperature. After they were washed, the sections were reacted with Alexa Fluor 488-labeled secondary goat anti-rabbit IgG antibodies. To stain cell nuclei, the sections were incubated with 25 ug/mL of 4′-6-diamidino-2-phenylindole (DAPI) in PBST for 30 min. Fluorescence images were captured of the right area of substantia nigra and analyzed using a confocal microscope (Leica, Solms, Germany).

### Western blot immunoassay

Substantia nigra was dissected from isolated brain tissue (9 mice per group) and then homogenized in PRO-PREP (iNtRON Biotechnology Inc., Seongnam, Korea) containing protease inhibitor. The tissue lysates were loaded on 6–15% polyacrylamide gels and separated by SDS-PAGE. After transfer, the membranes were blocked in 3% BSA or 5% skim milk dissolved in TBST for 1 h and then incubated overnight at 4 °C with primary antibodies. The membranes were washed in TBST and incubated with the appropriate secondary antibodies for 1 h at room temperature. The proteins on the membranes were detected using a SuperSignal West Femto Maximum Sensitivity Substrate kit (Thermo Scientific, Pierce Biotechnology, Rockford, Illinois, USA) and an LAS-4000 mini system (Fujifilm, Tokyo, Japan). The following primary antibodies were used: tyrosine hydroxylase (TH), α-synuclein, microtubule-associated protein 1 light chain 3 (LC3), sequestosome1/p62 (p62), mechanistic target of rapamycin (mTOR), AMP-activated protein kinase (AMPK), phosphoinositide 3-kinase (PI3K), unc-51-like kinase 1 (ULK) (all Cell Signaling Technology, MA, USA), and brain-derived neurotrophic factor (BDNF) (Sigma-Aldrich). The data were normalized to the intensity of GAPDH using Multi Gauge software (Fujifilm).

### Statistical analysis

All data were analyzed by one-way and two-way ANOVA. Differences between groups were considered significant at the *p* < 0.05 level after appropriate Bonferroni correction for multiple comparisons. The results are presented as the mean ± S.E.M.

## Electronic supplementary material

Dataset 1
