# Supplementary material for: Anodal transcranial direct current stimulation prevents methyl-4-phenyl-1,2,3,6-tetrahydropyridine (MPTP)-induced neurotoxicity by modulating autophagy in an in vivo mouse model of Parkinson’s disease
Source: Sci Rep. 2018 Oct 11;8:15165. doi: 10.1038/s41598-018-33515-7 (PMC6181991; doi:10.1038/s41598-018-33515-7)
Supplement: Supplementary file 1 — Dataset 1 [file 41598_2018_33515_MOESM1_ESM.doc]

**Supplementary Information**

Supplementary Figures 1-5

Supplementary method and result

**Anodal transcranial direct current stimulation prevents methyl-4-phenyl-1,2,3,6-tetrahydropyridine (MPTP)-induced neurotoxicity by modulating autophagy in an *in vivo* mouse model of Parkinson’s disease.**

Sang-Bin Lee, Hee-Tae Kim, Hyun Ok Yang, Wooyoung Jang

**Supplementary Figures**


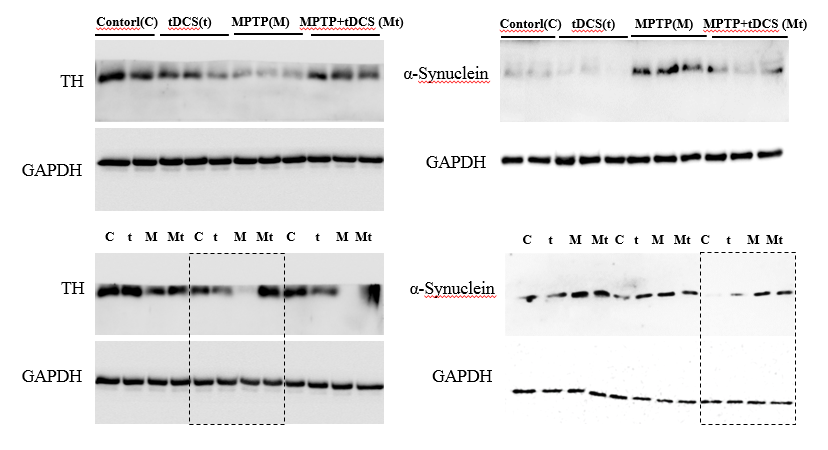


**Supplementary Figure 1.** Full uncropped scans of TH and α-Synuclein western blots shown in Figure 2 (Dashed box)

**
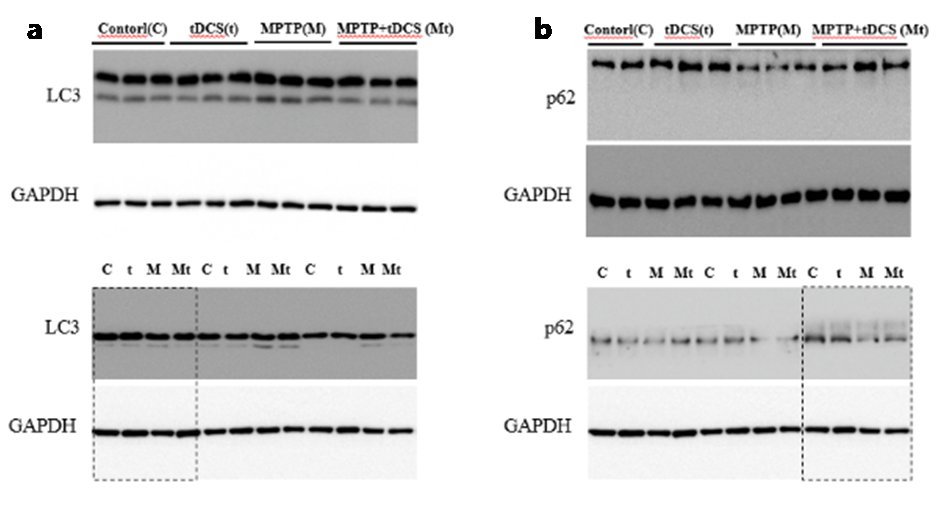
**

**Supplementary Figure 2.** Full uncropped scans of LC3(a) and p62(b) western blots shown in Figure 3 (Dashed box)


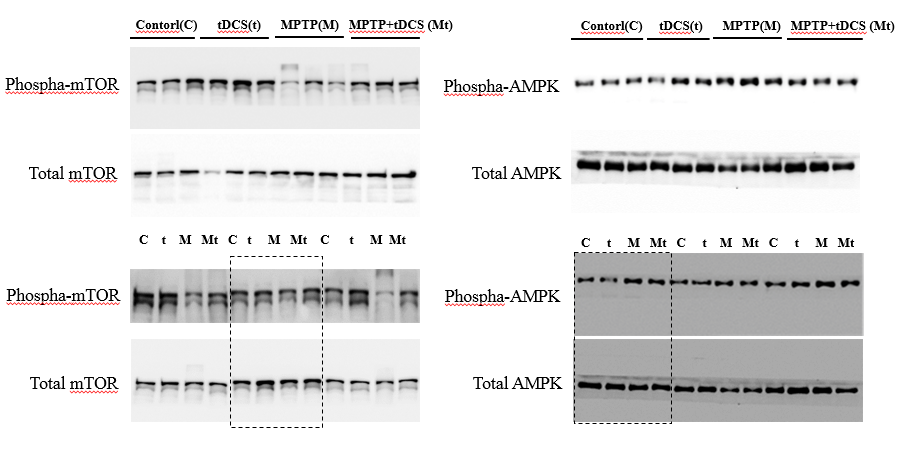


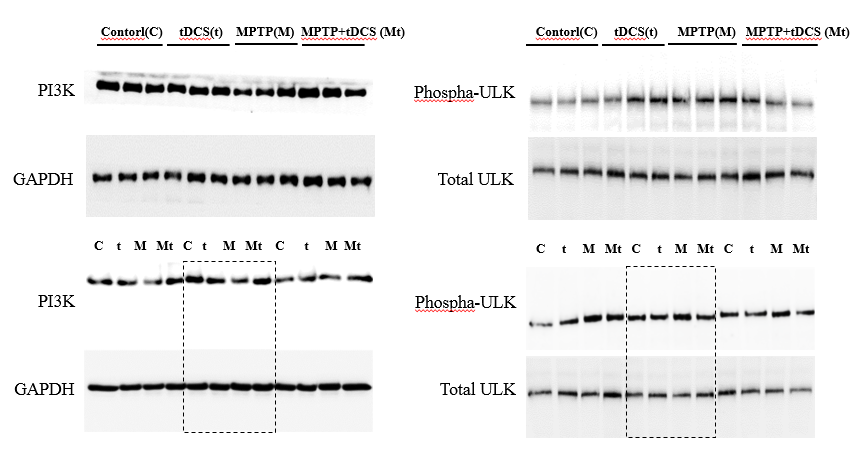


**Supplementary Figure 3.** Full uncropped scans of mTOR, AMPK, PI3K and ULK western blots shown in Figure 4 (Dashed box)


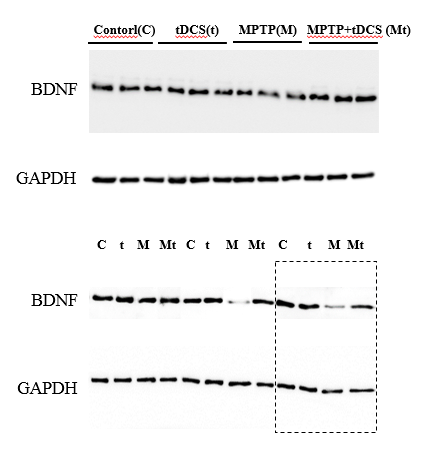


**Supplementary Figure 4.** Full uncropped scans of BDNF western blots shown in Figure 5 (Dashed box)


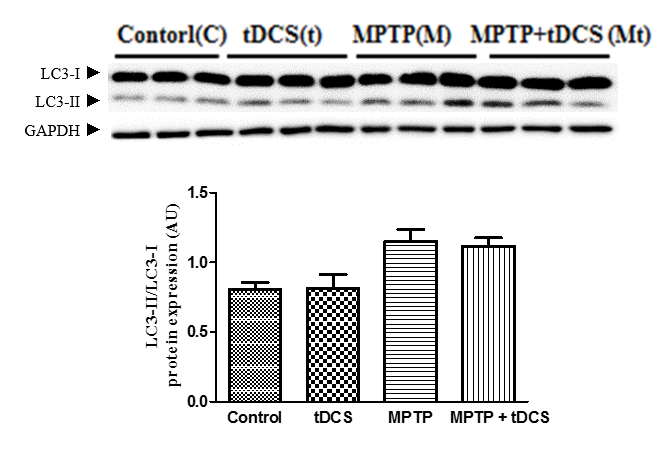


**Supplementary Figure 5**. Full uncropped scans of LC3 western blot applied by cathodal stimulation.

Effect of cathodal tDCS on LC3 protein expression in MPTP-treated mice. Western blotting was performed to measure the ratio of LC3-II and LC3-I. The ratio of the LC3-II and LC3-I bands was evaluated by densitometric analysis(n= 3). All values are shown as the mean ± S.E.M.

**Supplementary method**

For cathodal transcranial brain stimulation, mice were anesthetized with ketamine and xylazine (55 mg/kg and 7 mg/kg) and than custom-made plastic tube was placed on the skull of left M1 cortex area, which tube was attached to the skull by nontoxic dental cement. After five days recovery, tDCS was daily applied for 30 min using a constant current stimulator at 0.1 mA by a cathodal electrode 30 min after MPTP treatment for consecutive five days (Control and tDCS groups were not treated MPTP). Similar with anodal stimulation, cathodal electrode [3.1 mm2] was inserted into the custom-made plastic tube placed on the left M1 cortex area and the anode [11 cm2] was put between the shoulders. After five days tDCS with or without MPTP treatment, mice were sacrificed and the each brain was taken and than stored at -80℃ for western blotting.

**Supplementary result**

To determine the effects of cathodal tDCS on MPTP-induced autophagy, we measured the ratio of LC3-II/LC3-I in the mouse SNpc. The ratio of LC3-II/LC3-I was 1.4-fold higher in the MPTP group than was observed in the sham group. Cathodal tDCS did not affect the increased ratio of LC3-II/LC3-I (2-way ANOVA, tDCS: F = 0.0281, P = 0.8710; MPTP: F= 18.31, P = 0.0027; interaction: F = 0.07553, P = 0.7904).
